# Application of machine reading comprehension techniques for named entity recognition in materials science

**DOI:** 10.1186/s13321-024-00874-5

**Published:** 2024-07-02

**Authors:** Zihui Huang, Liqiang He, Yuhang Yang, Andi Li, Zhiwen Zhang, Siwei Wu, Yang Wang, Yan He, Xujie Liu

**Affiliations:** School of Biomedical and Pharmaceutical Sciences, Guangdong University of Technology, Guangzhou, 510006 China

**Keywords:** Text mining, Materials science, Named entity recognition, Machine reading comprehension

## Abstract

Materials science is an interdisciplinary field that studies the properties, structures, and behaviors of different materials. A large amount of scientific literature contains rich knowledge in the field of materials science, but manually analyzing these papers to find material-related data is a daunting task. In information processing, named entity recognition (NER) plays a crucial role as it can automatically extract entities in the field of materials science, which have significant value in tasks such as building knowledge graphs. The typically used sequence labeling methods for traditional named entity recognition in material science (MatNER) tasks often fail to fully utilize the semantic information in the dataset and cannot effectively extract nested entities. Herein, we proposed to convert the sequence labeling task into a machine reading comprehension (MRC) task. MRC method effectively can solve the challenge of extracting multiple overlapping entities by transforming it into the form of answering multiple independent questions. Moreover, the MRC framework allows for a more comprehensive understanding of the contextual information and semantic relationships within materials science literature, by integrating prior knowledge from queries. State-of-the-art (SOTA) performance was achieved on the Matscholar, BC4CHEMD, NLMChem, SOFC, and SOFC-Slot datasets, with F1-scores of 89.64%, 94.30%, 85.89%, 85.95%, and 71.73%, respectively in MRC approach. By effectively utilizing semantic information and extracting nested entities, this approach holds great significance for knowledge extraction and data analysis in the field of materials science, and thus accelerating the development of material science.

**Scientific contribution**

We have developed an innovative NER method that enhances the efficiency and accuracy of automatic entity extraction in the field of materials science by transforming the sequence labeling task into a MRC task, this approach provides robust support for constructing knowledge graphs and other data analysis tasks.

**Supplementary Information:**

The online version contains supplementary material available at 10.1186/s13321-024-00874-5.

## Introduction

The field of materials science has witnessed a significant surge in research and literature in recent years. While scientific publications offer valuable and reliable data, the manual analysis of a vast number of papers to extract essential information for materials can be an arduous undertaking. The manual extraction of this information is time-consuming, impeding researchers’ ability to access the necessary information. Emerging technologies in natural language processing (NLP) offer promising solutions to the process of extracting relevant information from scientific literature. Among them, automatically recognizing named entities in a given text is an important task in the field of NLP. In materials science, identification of various materials, compounds, elements, and other entities is crucial for extracting and transforming material science knowledge from unstructured texts. However, the task of identifying named entities in materials science (MatNER) [1–5] is extremely challenging because there are multiple entities in the materials science literature and their complex combinations, such as acronyms, misspellings, synonyms of compound names, etc.

In the early stages, named entity recognition (NER) mainly relied on rule-based and handcrafted feature methods [3, 6–10]. These methods required manual definition of rules and feature templates, and had high requirements for domain knowledge. However, due to the complexity and limitations of rules and features, these methods had poor adaptability to different languages and domains. As machine learning has gained popularity, statistical and machine learning techniques have been increasingly utilized in NER. These approaches leverage annotated datasets to train models, enabling them to learn the statistical patterns and contextual information associated with entities in text. Common machine learning algorithms include Hidden Markov Models (HMM) [11], Conditional Random Fields (CRF) [12], and deep learning models. Deep learning has made significant progress in the field of NER [13–17]. Deep learning models can automatically learn text feature representations, extracting and classifying information through multi-layer neural networks. For example, Bidirectional Long Short-Term Memory networks (BiLSTM) [14] with Recurrent Neural Networks (RNNs) [18] can capture the contextual dependencies between entities, improving the accuracy of NER. Furthermore, the emergence of large-scale pre-trained language models like ELMo [19] and BERT [20] has greatly benefited NER. Due to its significant performance, pre-training BERT on large corpora and fine-tuning on target datasets has become a mainstream approach. In the field of materials science, Gupta et al. [21] used MatSciBERT, i.e., BERT pre-trained on materials science corpus, to recognize material science entities, and their method achieved SOTA performance on multiple materials science datasets. The ability of deep learning methods to automatically learn features results in more competitive performance compared to feature engineering methods.

Existing methods typically approach the MatNER task by treating it as a sequence labeling problem. This involves training a model to assign labels to individual tokens in a given sequence. However, these methods have limitations in effectively capturing semantic information and addressing the nested entity problem. Motivated by the recent trend of transforming NLP tasks as machine reading comprehension (MRC) tasks [22–27], a MatSciBERT-MRC method based on the MRC framework was proposed in this study. In the MRC framework, each type of material science entity can be encoded through language queries and extracted in the given context by answering these queries, thus more effectively utilizing the information in the training data and improving the generalization ability of the model. Recent studies have converted various NLP tasks into MRC tasks. For instance, Levy et al. [23] proposed a method to cast the relation extraction task as a QA task by parameterizing each relation type R(x,y) as a question Q(x), with y being the answer. Similarly, McCann et al. [24] achieved competitive performance by uniformly implementing 10 different NLP tasks using a question answering framework. In the field of Named Entity Recognition (NER), Li et al. [26] applied BERT for entity recognition under the MRC framework in texts from regular domains, while Sun et al. [27] attained significant performance in texts from the biomedical domain.

To our knowledge, no specific study has focused on NER in materials science under the MRC framework. Herein, we aim to identify entities in materials science, which differs from previous research [26, 27]. Additionally, the impact of different MRC strategies on the MatNER task is explored. The performance of MatSciBERT-MRC was evaluated on five public materials science datasets, and a comparison was made with traditional sequence labeling models. Experimental results showed that MatSciBERT-MRC has good performance in detecting various material names, compounds, elements, etc., achieving the latest SOTA performance. A powerful tool is provided to material science researchers by this research, enabling them to handle large-scale material science literature and data more accurately and efficiently. Accurately identifying and extracting key information can accelerate the material research process and provide more possibilities for material design and discovery.

## Methodology

### Datasets construction

The input to a traditional sequence annotation task is a sequence $X=\{{x}_{1},{x}_{2},... , {x}_{N}\}$, where ${x}_{i}$ represents the i-th word or label in the sequence. In this study, the labeled NER data needs to be transformed into triples of (Context, Query, and Answer). The Context is a given input sequence $X$, the Query is a query sentence designed based on that sequence $X$, and the Answer is the scope of the target entity. In the MRC task, the construction of the query sentence ${Q}_{y}$ to obtain relevant information is required. Specifically, for each type of entity, we can use keywords or phrases associated with label $y$ and combine them into a query sentence. The length of the query sentence can be determined based on the specific requirements of the task.

### Query generation

The generation of queries is recognized as a crucial process as it encompasses prior knowledge of labels, which ultimately influences the final results of MatNER tasks. In this study, the creation of queries relied upon annotation guidelines as references. These guidelines are composed of instructions provided by dataset producers to annotators, enabling them to effectively describe label categories. It is essential that these guidelines be expressed in a broad and precise manner in order to eliminate any ambiguity. Table 1 presents examples of queries we constructed in Matscholar [1] dataset.

**Table 1:** Examples of constructed queries

Entity type	Query
MAT	Any inorganic solid or alloy, any non-gaseous element
SPL	Names for crystal structures/phases
DSC	Special descriptions of the type/shape of the sample
PRO	Anything measurable that can have a unit and a value
APL	Any high-level application such as photovoltaics, or any specific device such as field-effect transistor
CMT	Any method used to characterize a material
SMT	Any technique for synthesizing a material

### Model details

**Fig. 1:**
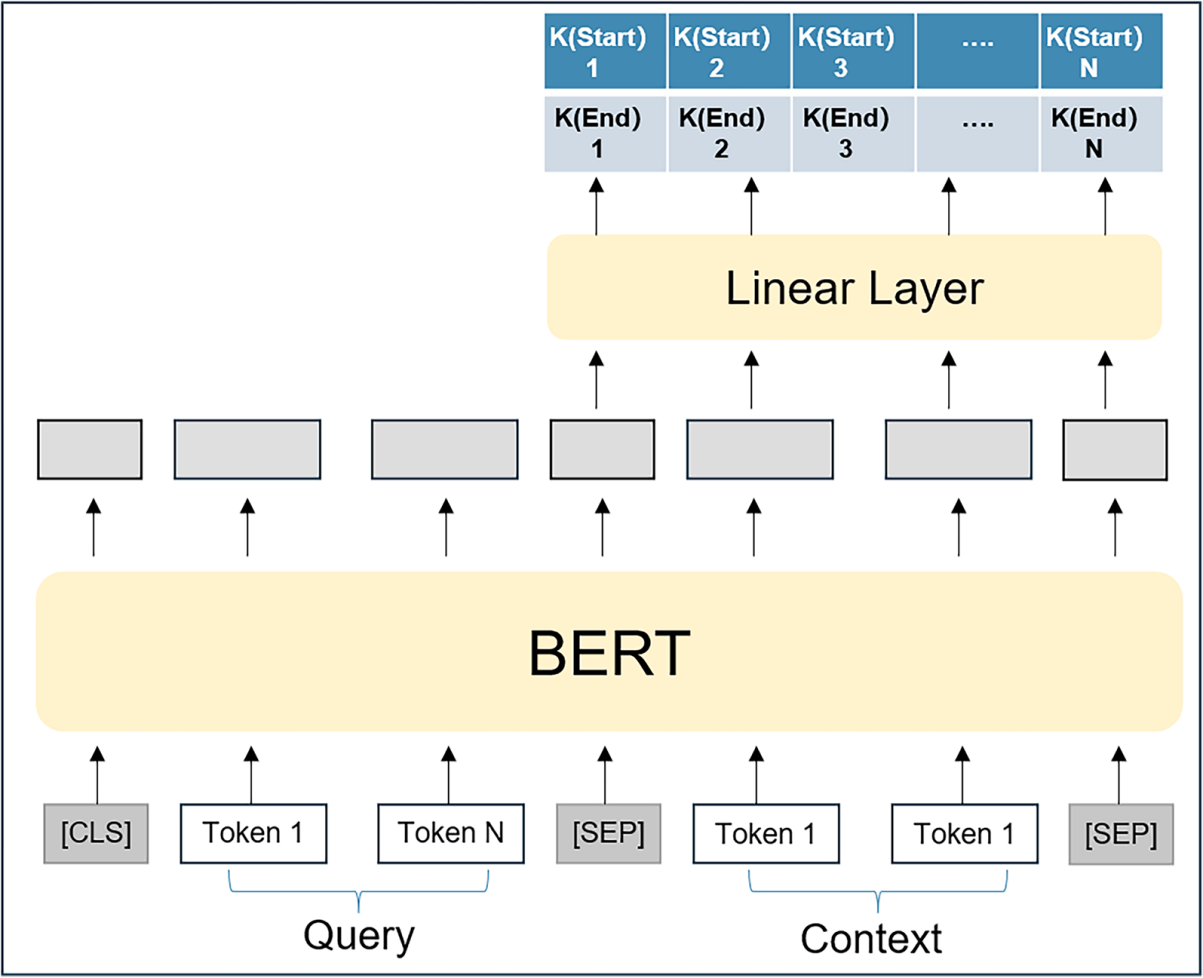
Using BERT to perform MatNER in the MRC framework

In this study, BERT [20] was used as the model backbone, along with MatsciBERT [21] as the model weights, to identify entities in the field of materials science. Figure 1 depicts the implementation of the MatNER task using BERT in the MRC framework. Initially, the combined sequence{[CLS],${q}_{1}$,${q}_{2}$,…,${q}_{m}$, [SEP],${x}_{1}$,${x}_{2}$,…,${x}_{n}$} is formed by concatenating the query ${Q}_{y}$ with the sequence X, the special tokens [CLS] and [SEP] are used to represent the start and end positions of the labels. These tokens are combined with the input sequence and fed into the BERT model, which received the combined string and outputs the context representation. Since we only require context predictions, query representations can be removed as they are not part of the target for model prediction.

In the MRC framework, there are two prevalent approaches to select spans. One strategy involves employing a pair of n-class classifiers, where one is responsible for predicting the starting index and the other for predicting the ending index. These classifiers can extract features using pre-trained models like BERT and output an n-dimensional probability distribution, indicating the probability of each position being the start or end position. Then, the highest probability start and end positions can be selected to form a span. Another approach involves the utilization of two binary classifiers, wherein one classifier is responsible for predicting the start index of each position, while the other classifier is responsible for predicting the end index of each position. Similarly, these binary classifiers can extract features using pre-trained models and output a binary probability distribution, indicating the probability of each position being the start or end position. This approach enables the output of multiple start and end indexes, catering to a given context and specific query, making it possible to extract all relevant entities based on the query. The second approach is utilized in this study and a detailed explanation of its workings is provided.

For the prediction of start index, softmax is used to get whether the token is start index using the following equation:

1 $${K}_{start} = linear(L{Q}_{start} ) \in {R}^{N\times 2}$$

where ${Q}_{start}$ is the weight to be learned and the probability distribution of each index as the starting position of the entity is represented by each row of ${K}_{start}$.

The model then predicts the probability of each token being the corresponding end index, using the following formula:

2 $${K}_{end} = linear\left(L{Q}_{end}; softmax\left({K}_{start}\right)\right)\in {R}^{N\times 2}$$

In order to determine the ending position of an entity for a given query, we introduce the weight ${Q}_{end}$, which is to be learned. The probability distribution of each index as the ending position of the entity is represented by each row of ${K}_{end}$.

For each given X, there may exist multiple possible start and end indices. Simply matching them based on proximity is not a reasonable approach. Therefore, by applying argmax to the output matrix for each row of ${K}_{start}$ and ${K}_{end}$, we are able to obtain all possible start and end indices. This approach allows us to identify the most probable start and end indices, as determined by the following formula:

3 $${I}_{start} =\{i|argmax({K}_{start}^{i}) = 1, i = 1, 2, \cdots , N\}$$

4 $${I}_{end} =\{j|argmax({K}_{end}^{j}) = 1, j = 1, 2, \cdots , N\}$$

Superscripts i and j are used to indicate the i-th and j-th rows of the matrix, respectively.

### Datasets and experiment settings

Five different NER datasets were considered, i.e. BC4CHEMD [28], Matscholar [1], NLMChem [29], SOFC-Slot and SOFC [30], to represent various text sources and questions related to materials science. Table 2 displays the statistics of all datasets used in this study, which were selected because they are publicly available and ensured a comprehensive evaluation of the proposed method.

**Table 2:** Statistics on datasets

Dataset	Annotation	Sentences	Entity types
BC4CHEMD	79,842	89,679	1
Matscholar	–	–	7
NLMChem	34,404	40,467	1
SOFC	5095	9466	4
SOFC-Slot	4179	9466	17

The experiments in this study were conducted using Python 3.8.12 and torch 1.12.1. Training of the models was performed on a single GTX 3060 GPU. Due to the computational complexity limitations, many previous works in the field of material science utilized the ${\text{BERT}}_{\text{BASE}}$ model. To ensure comparability with these works, all BERT models employed in this study were based on the ${\text{BERT}}_{\text{BASE}}$ [20] model, which consists of 12 transformer layers, a 768-dimensional hidden layer, and a 12-head multi-head attention mechanism. For specific details on the hyperparameters used in the experiments, please refer to Table 3.

**Table 3:** The detailed hyper-parameters of MatSciBERT-MRC

Dataset	seq_len	bs	lr	Loss
BC4CHEMD	512	8	2e-5	Focal
Matscholar	512	16	2e-5	Focal
NLMChem	512	8	2e-5	Focal
SOFC	512	8	2e-5	Focal
SOFC-Slot	512	8	2e-5	Focal

### Evaluation metrics

In the experimental phase, the F1-score was employed as the metric to assess the overall performance of the model. Furthermore, precision and recall were utilized to evaluate the model’s capability in accurately identifying positive and negative samples.

Precision can be defined as the ratio of correctly identified positive values, also known as true values, to the total number of identified positive values.

5 $$P=\frac{TP}{TP+FP}$$

Recall is the ratio between the predicted true value and the actual labeled result.

6 $$R=\frac{TP}{TP+FN}$$

F1-score is the harmonic mean of precision and recall.

7 $$F1=\frac{2PR}{P+R}$$

## Result and discussion

### The effect of different BERT models on NER performance

To investigate the effects of different BERT models on NER performance, the performance of MatSciBERT [21], BioBERT [31], and SciBERT [32] was evaluated for the NER task. The aim was to determine which BERT model would yield the best performance for NER in materials science. The effect of different BERT models on NER performance is illustrated in Table 4. Overall, MatsciBERT achieved higher scores than BioBERT and SciBERT on all the datasets (p < 0.05). Unlike SciBERT, MatSciBERT has been trained using a large corpus of texts from the field of materials science, covering multiple research fields, journals, and data sources, encompassing a broad body of materials science knowledge and domain-specialized terminology. This gives MatSciBERT greater adaptability and accuracy when working with materials science-related texts.

**Table 4:** Performance comparison for different BERT Models

Dataset	Model	Mean ± std	Max
Matscholar	BioBERT	84.81 ± 0.35	85.39
	SciBERT	85.97 ± 0.36	86.41
	MatSciBERT	**87.81 ± 0.16**	**87.97**
BC4CHEMD	BioBERT	92.36 ± 0.12	92.51
	SciBERT	92.18 ± 0.08	92.29
	MatSciBERT	**92.64 ± 0.23**	**92.96**
NLMChem	BioBERT	82.12 ± 0.30	82.42
	SciBERT	82.87 ± 0.38	83.33
	MatSciBERT	**83.87 ± 0.35**	**84.23**
SOFC	BioBERT	81.15 ± 0.23	81.47
	SciBERT	82.85 ± 0.60	83.52
	MatSciBERT	**84.15** ± 0.11	**84.26**
SOFC-Slot	BioBERT	60.02 ± 0.61	60.83
	SciBERT	66.25 ± 0.14	66.44
	MatSciBERT	**67.51 ± 0.54**	**68.12**

The bold marking indicates the highest F1 score in the comparison of different BERT Models

These experimental results indicate that there is a significant difference between the scientific literature in the materials domain pre-trained by MatSciBERT and the scientific literature in the biomedical domain pre-trained by BioBERT. It becomes evident that each scientific discipline presents substantial variation in terms of ontology, vocabulary, and domain-specific symbols. Therefore, the pre-trained corpus plays a pivotal role in determining the model’s performance, and the utilization of MatSciBERT, trained on an extensive collection of materials science publications, proves to be more fitting for our MatNER task in this experiment.

### MRC vs sequence labeling frameworks

A detailed comparison of the performance of BERT models with MRC framework and sequence tagging framework was conducted. The performance comparison between different models is presented in Table 5. In the MatSciBERT-Softmax, the classification of each token in the sequence is accomplished by utilizing the Softmax function on the MatSciBERT output. MatSciBERT-CRF learns the constraint relationships between labels through CRF to ensure the rationality of the predicted label sequence, thereby obtaining the best sequence annotation results. MatSciBERT-BiLSTM-CRF uses BiLSTM-CRF to enhance the learning ability of the context, enabling the model to better learn semantic information in the context. Among the three sequence labeling frameworks (i.e. MatSciBERT-CRF, MatSciBERT-BiLSTM-CRF, and MatSciBERT-Softmax), MatSciBERT-CRF achieves the best performance in all four datasets in the traditional sequence annotation model. It can be inferred that the CRF possesses the ability to acquire the interdependent connection between labels. This capability ultimately guarantees the rationality of the predicted label sequence and significantly enhances the accuracy of entity recognition. However, for BC4CHEMD dataset, the performance of MatSciBERT-BiLSTM-CRF model is the best among the three series of annotation models, which may be due to the relatively large items in BC4CHEMD dataset. The MatSciBERT-BiLSTM-CRF model can learn more contextual semantic information from the dataset.

**Table 5:** Performance comparison for different models

Dataset	Model	Mean ± std	Max
Matscholar	MatSciBERT-Softmax	87.90 ± 0.17	88.08
	MatSciBERT-CRF	88.45 ± 0.23	88.75
	MatSciBERT-BiLSTM-CRF	88.35 ± 0.09	88.48
	MatSciBERT-MRC	**89.59 ± 0.05**	**89.64**
BC4CHEMD	MatSciBERT-Softmax	93.36 ± 0.11	93.51
	MatSciBERT-CRF	93.45 ± 0.08	93.56
	MatSciBERT-BiLSTM-CRF	93.60 ± 0.14	93.77
	MatSciBERT-MRC	**94.18 ± 0.10**	**94.30**
NLMChem	MatSciBERT-Softmax	84.17 ± 0.23	84.46
	MatSciBERT-CRF	84.37 ± 0.32	84.81
	MatSciBERT-BiLSTM-CRF	84.19 ± 0.38	84.67
	MatSciBERT-MRC	**85.79 ± 0.09**	**85.89**
SOFC	MatSciBERT-Softmax	84.03 ± 0.13	84.18
	MatSciBERT-CRF	84.72 ± 0.27	85.04
	MatSciBERT-BiLSTM-CRF	84.24 ± 0.17	84.46
	MatSciBERT-MRC	**85.71 ± 0.20**	**85.95**
SOFC-Slot	MatSciBERT-Softmax	68.78 ± 0.48	69.35
	MatSciBERT-CRF	70.22 ± 0.09	70.34
	MatSciBERT-BiLSTM-CRF	69.61 ± 0.11	69.72
	MatSciBERT-MRC	**71.58 ± 0.14**	**71.73**

The bold marking indicates the highest F1 score in the comparison of all model architectures, while the underline marking indicates the best F1 score among models excluding the MRC architecture

Unlike the above three methods, MatSciBERT-MRC turns the MatNER task into a machine reading comprehension problem and predicts the answer span ${x}_{start, end}$ based on the input sequence $X$ and query statement ${Q}_{y}$. As shown in Table 5, compared with sequence tagging methods, MatSciBERT-MRC improves a substantial enhancement to the performance of the MatNER task (p < 0.05). By encoding crucial prior knowledge into the query, the MRC effectively mitigates the problem of sparse tagging, corpus size or sentence length, leading to more improvements on all the datasets. The experimental results unequivocally showcase the superior entity identification capabilities of BERT within the MRC framework compared to the sequence tagging framework, particularly in the domain of material science.

### The effect of different MRC strategies on NER performance

The influence of different span prediction strategies was also evaluated in this study. Specifically, the effect of end_index information in MatSciBERT-MRC model on the NER performance was investigated. To assess this, a baseline model called MatSciBERT-MRC-base was designed and its performance was compared to that of MatSciBERT-MRC. In implementation, MatSciBERT-MRC-base only replaced ${K}_{end}$ of MatSciBERT-MRC described in front with the following formula, while keeping everything else unchanged:

8 $${K}_{end} = linear\left(L{Q}_{end}\right)\in {R}^{N\times 2}$$

A performance comparison between these two models is presented in Table 6. It can be observed that both models have competitive average F1 scores. Overall, MatSciBERT-MRC outperformed MatSciBERT-MRC-base on four out of five datasets (p < 0.05). This advantage is likely because the model considers the start index when predicting the end index, allowing for more accurate boundary prediction of entities. The start index provides context, helping the model determine the most likely end position of an entity, thereby reducing errors in boundary prediction. Additionally, independently predicting the start and end indices can lead to invalid spans, such as the end index being before the start index or spans that do not correspond to valid entities. However, the base model performed better than MatSciBERT-MRC models in the BC4CHEMD dataset, possibly because the entity structure in this dataset is relatively simple, allowing the baseline model to better capture this simple structure.

**Table 6:** Performance comparison for different end index strategies

Dataset	Model	Mean ± std	Max
Matscholar	MatSciBERT-MRC-base	88.73 ± 0.09	88.84
	MatSciBERT-MRC	**89.59 ± 0.05**	**89.64**
BC4CHEMD	MatSciBERT-MRC-base	**94.25 ± 0.16**	**94.45**
	MatSciBERT-MRC	94.18 ± 0.10	94.30
NLMChem	MatSciBERT-MRC-base	84.95 ± 0.08	85.04
	MatSciBERT-MRC	**85.79 ± 0.09**	**85.89**
SOFC	MatSciBERT-MRC-base	85.08 ± 0.16	85.26
	MatSciBERT-MRC	**85.71 ± 0.20**	**85.95**
SOFC-Slot	MatSciBERT-MRC-base	70.32 ± 0.44	70.91
	MatSciBERT-MRC	**71.58 ± 0.14**	**71.73**

The bold marking indicates the highest F1 score in the comparison of different end index strategies

Based on the experimental findings, it has been observed that the model's performance can be influenced to a certain degree by the implementation of different end_index functions. Furthermore, considering the start index during the prediction of the end index has been found to enhance the overall performance of the model.

Due to the extensive data processing required in the early stage of MRC model, it has led to a decrease in the number of entities and an imbalance in labels. To address these issues, this study investigates the impact of different loss functions on model performance, comparing Focal Loss, CrossEntropy Loss, and Label Smoothing. Focal Loss tackles class imbalance by adjusting the weights of samples, with particular focus on difficult-to-classify samples. In contrast, CrossEntropy Loss measures the accuracy of the model by calculating the difference between predicted results and true labels. Label Smoothing introduces some noise to make the labels relatively soft, thereby alleviating overfitting to the training data.

In this experiments, three distinct loss functions were employed to evaluate the performance of the model. The results in Table 7 demonstrate that using Focal Loss as the loss function leads to a certain improvement in model performance (p < 0.05). This can be attributed to Focal Loss effectively addressing the issue of class imbalance, thereby enhancing the model’s ability to classify difficult samples. In conclusion, selecting an appropriate loss function is crucial for improving the performance of MRC models. When dealing with class imbalance, Focal Loss may be an effective choice.

**Table 7:** Performance comparison for different Losses

Dataset	Loss	Mean ± std	Max
Matscholar	Focal	**89.59 ± 0.05**	**89.64**
	CrossEntropy	87.95 ± 0.06	88.02
	Label smoothing	88.00 ± 0.13	88.15
BC4CHEMD	Focal	**94.18 ± 0.10**	**94.30**
	CrossEntropy	94.05 ± 0.11	94.20
	Label smoothing	94.05 ± 0.14	94.24
NLMChem	Focal	**85.79 ± 0.09**	85.89
	CrossEntropy	85.74 ± 0.21	**86.01**
	Label smoothing	85.19 ± 0.05	85.26
SOFC	Focal	**85.71 ± 0.20**	**85.95**
	CrossEntropy	84.45 ± 0.25	84.76
	Label smoothing	85.02 ± 0.05	85.09
SOFC-Slot	Focal	**71.58 ± 0.14**	**71.73**
	CrossEntropy	71.01 ± 0.10	71.12
	Label smoothing	71.23 ± 0.11	71.36

The bold marking indicates the highest F1 score in the comparison of different Loss

Our findings show that incorporating appropriate loss functions and span prediction strategies can significantly improve the performance of the model on imbalanced datasets.

### The effect of different query constructs on NER performance

The structure of the query plays a crucial role in determining the final outcomes. In this section, different approaches to query construction and their implications was explored. The label “MAT” in the Matscholar dataset was used as an example. Several common methods for query construction were employed, including:

- Keywords: The query describes the label using keywords. For example, the query for the label MAT is “inorganic material”.
- Rule-based template filling: Queries are generated using templates. The query for the label MAT is “Which inorganic material is mentioned in the text?”.
- Wikipedia: Queries are constructed using Wikipedia definitions. The query for the label MAT is “Materials made from inorganic substances alone or in combination with other substances”.
- Synonyms: Words or phrases that possess identical or closely similar meanings to the original keyword extracted from the Oxford Dictionary. The query for the label MAT is “Inorganic material”.
- Keywords + Synonyms: Keywords are combined with their synonyms.
- Annotation guideline annotation: This is the method we used in this paper. The query for the label MAT is “Look up any inorganic solids or alloys, any non-gaseous elements.”

The experimental results of our MatNER on the Matscholar dataset are presented in Table 8. The BERT-MRC model performs better than the BERT-Tagger model in all settings. The Annotation Guideline Notes method outperforms other methods because it provides clearer and more detailed label definitions (p < 0.05). These guidelines typically include instructions provided by dataset producers to annotators, making the label category descriptions more explicit and specific, thereby reducing ambiguity and errors in the annotation process. In contrast, Wikipedia falls short in comparison to Annotation Guideline Notes. This can be attributed to the relatively general definitions provided by Wikipedia, which may not precisely align with the specific data annotations required. These findings highlight the importance of query construction in MatNER tasks. The use of carefully designed queries can significantly improve the performance of MatNER models.

**Table 8:** Performance comparison for different Query constructs

Model	Mean ± std	Max
BERT-Tagger	88.45 ± 0.25	88.75
Keywords	89.07 ± 0.05	89.13
Rule-based template filling	88.81 ± 0.14	88.98
Wikipedia	88.72 ± 0.24	88.91
Synonyms	89.01 ± 0.04	89.07
Keywords + Synonyms	89.17 ± 0.04	89.23
Annotation guideline annotation	**89.59 ± 0.05**	**89.64**

The bold marking indicates the highest F1 score in the comparison of different Query constructs

### Performance comparison with other methods

The main purpose of this work is to compare the proposed method with previous studies on five material science datasets. We observed a significant improvement in the performance of the material science dataset compared to the previous SOTA models. The F1 scores on the Matscholar, BC4CHEMD, NLMChem, SOFC and SOFC-Slot datasets were 89.64%, 94.30%, 85.89%, 85.95% and 71.73%, respectively, which represent an improvement of + 0.89%, + 2.55%, + 1.08%, + 0.91%, and + 1.39% over the previous SOTA performances.

These results demonstrate the superior performance of our method in the field of material science, surpassing previous benchmarks. This improvement is crucial for information extraction and entity recognition tasks in material science. Through experiments on these five datasets, the robustness, generality, and effectiveness of our method have been validated across multiple datasets and different scenarios (Table 9). In addition, to verify the effectiveness of our model, we have also conducted a five-fold cross-validation on the dataset. For more details, please refer to Table S2 in the supporting information.

**Table 9:** Performance comparison with other existing methods

Dataset	Model	Precision	Recall	F1
Matscholar	BiLSTM-CRF [1]	–	–	87.09
	BERT-base [33]	81.00	81.90	81.40
	MatSciBERT-CRF	88.01	89.51	88.75
	MatSciBERT-MRC	90.39	88.89	**89.64**
BC4CHEMD	tmChem [3]	89.09	85.75	87.39
	BiLSTM-CRF [13]	92.29	90.01	91.14
	CollaboNet [34]	90.78	87.01	88.85
	HanPaNE + P [35]	92.80	92.30	92.60
	MatSciBERT-CRF	93.08	94.05	93.56
	MatSciBERT-MRC	93.96	94.64	**94.30**
NLMChem	TaggerOne [36]	72.40	61.50	66.50
	BlueBERT + MTCR [37]	81.00	71.10	75.70
	BioNER-Cache [38]	84.32	85.27	84.79
	MatSciBERT-CRF	82.44	87.32	84.81
	MatSciBERT-MRC	89.56	82.52	**85.89**
SOFC	CRF [30]	–	–	60.30
	BiLSTM word2vec [30]	–	–	56.30
	BiLSTM BERT-base [30]	–	–	79.10
	BERT-base [30]	–	–	78.40
	MatSciBERT-CRF	85.22	84.87	85.04
	MatSciBERT-MRC	87.17	84.76	**85.95**
SOFC-Slot	CRF [30]	–	–	45.30
	BiLSTM BERT-base [30]	–	–	63.30
	BiLSTM SciBERT [30]	–	–	67.80
	BERT-base [30]	–	–	63.40
	MatSciBERT-CRF	71.14	69.55	70.34
	MatSciBERT-MRC	72.18	71.28	**71.73**

The [bold, underline] notation indicates the highest F1 score among all models compared

### Case study

A comprehensive case study was conducted to further investigate the distinctions between MatSciBERT-MRC and MatSciBERT-CRF. The outcomes of the case study are presented in Table 10. Based on the case studies of Matscholar, BC4CHEMD, and SOFC-Slot, we can observe that the MatSciBERT-MRC model provides an accurate demarcation of the boundaries of entities, such as “UV-light illumination”, “docosahexaenoic acids”, “ceria-based ceramics”. It can therefore be inferred that MatSciBERT-MRC model is able to successfully identify words and phrases related to entity categories and provide accurate boundary information. In contrast, MatSciBERT-CRF model has limitations in accurately determining boundary information, which may be attributed to the difficulties encountered by the CRF model in handling complex syntactic structures and boundary information. Furthermore, in the case studies of NLMChem and SOFC, we can also clearly observe that the MatSciBERT-MRC model is able to identify “ceramic cell” entities which MatSciBERT-CRF fails to capture and corrected MatSciBERT-CRF's misidentification of “Immunocytochemistry (ICC)” entities. This further validates the superiority of the MatSciBERT-MRC model in entity recognition tasks.

**Table 10:** Representative results of case study

Dataset	Model	Sample
Matscholar	MatSciBERT-CRF	The UV**- light illumination** not only affects the morphology of the films …
	MatSciBERT-MRC	The **UV- light illumination** not only affects the morphology of the films …
BC4CHEMD	MatSciBERT-CRF	Fish contains both beneficial substances e.g. **docosahexaenoic** acids …
	MatSciBERT-MRC	Fish contains both beneficial substances e.g. **docosahexaenoic acids** …
NLMChem	MatSciBERT-CRF	**Immunocytochemistry(ICC)** was performed for leukocyte common …
	MatSciBERT-MRC	Immunocytochemistry(ICC) was performed for leukocyte common …
SOFC	MatSciBERT-CRF	Here the authors report a micro-monolithic ceramic cell design …
	MatSciBERT-MRC	Here the authors report a micro-monolithic **ceramic cell** design …
SOFC-Slot	MatSciBERT-CRF	Such as **ceria-based** ceramics for electrolyte and mixed ion–electron …
	MatSciBERT-MRC	Such as **ceria-based ceramics** for electrolyte and mixed ion–electron.
Other	MatSciBERT-CRF	The prepared **BWT-Pt catalysts** were used for aerobic oxidation reaction of alcohols …
	MatSciBERT-MRC	The prepared **BWT-Pt catalysts** were used for aerobic oxidation reaction of alcohols …

In addition, since our material dataset lacks nested cases, we artificially created nested cases to evaluate the performance of both models. MatSciBERT-CRF can only recognize “BWT-Pt catalysts” entities, while MatSciBERT-MRC can recognize “BWT-Pt” entities nested within “BWT-Pt catalysts” entities. MatSciBERT-MRC addresses the limitation of sequence annotation architectures and efficiently handling nested entities. These examples can be inferred that MatSciBERT-MRC excels at accurately identifying entity boundaries while mitigating label inconsistency and resolving entity nesting issues. This highlights the robustness and practicality of MatSciBERT-MRC in various scenarios related to material science information extraction. This advancement is not only pivotal for materials science but also has broader implications. For instance, this method can be adapted for named entity recognition in various domains such as chemistry and biosciences. In these fields, the MRC-based approach can effectively handle diverse texts, including those related to chemical synthesis, chemical property analysis, biological processes, etc.

## Conclusion

In summary, BERT in the MRC framework was employed to conduct named entity recognition in material science (MatNER) task. Compared to BERT in the sequence labeling framework, BERT (i.e., MatSciBERT) in the MRC framework can improve the performance in recognizing target entities. Moreover, the MRC framework has the advantage of incorporating prior knowledge, which can be effectively enhanced in performance through query design. The proposed approach achieves good SOTA performance on five MatNER datasets. The results demonstrate that utilizing BERT in the MRC framework with carefully designed queries can significantly improve the accuracy of MatNER models. The results clearly indicate that utilizing BERT in the MRC framework with thoughtfully designed queries can significantly improve the accuracy of MatNER models. By demonstrating the versatility and effectiveness of BERT in the MRC framework, our findings contribute to the development of more accurate and efficient natural language processing tools. These tools can be instrumental across a range of applications in materials science, chemistry, biosciences, and other fields, enabling precise extraction of named entities from different types of scientific texts.

### Supplementary Information

**Additional file 1: Table S1.** The query used by materials science datasets.

**Additional file 2:** Supplementary Information for fivefold cross-validation.

## Data Availability

The code of this study was written using PyTorch and Transformers library and is available at the GitHub repository https://github.com/huilkq/MatsciBERT_MRC, which also includes the code of MatsciBERT_MRC usage and data processing. The code and datasets for training our model can be found in this GitHub repository to ensure the reproducibility of this work. Additionally, all the pre-trained models and datasets used for fine-tuning are publicly available.
