# Supplementary material for: Application of machine reading comprehension techniques for named entity recognition in materials science
Source: J Cheminform. 2024 Jul 2;16:76. doi: 10.1186/s13321-024-00874-5 (PMC11220966; doi:10.1186/s13321-024-00874-5)
Supplement: Supplementary file 2 — Additional file 2: Supplementary Information for fivefold cross-validation. [file 13321_2024_874_MOESM2_ESM.docx]

**Supporting Information for**

Application of Machine Reading Comprehension Techniques for Named Entity Recognition in Materials Science

Zihui Huang ^a §^, Liqiang He ^a §^, Yuhang Yang ^a^, Andi Li ^a^, Zhiwen Zhang ^a^, Siwei Wu ^a^, Yang Wang ^a^, Yan He ^a^* and Xujie Liu ^a^*

^a^ School of Biomedical and Pharmaceutical Sciences, Guangdong University of Technology, Guangzhou 510006, China

* Corresponding authors: Email: [heyan129@gdut.edu.cn](mailto:heyan129@gdut.edu.cn) (Yan He); [liuxujie@gdut.edu.cn](mailto:liuxujie@gdut.edu.cn) (Xujie Liu)As we utilized publicly available datasets in our research, these datasets were pre-divided into training, validation, and test sets. To ensure consistency with prior studies and facilitate result comparability, we employed these pre-partitioned datasets for our analysis.

Simultaneously, to further validate the performance and reliability of our model, we conducted 5-fold cross-validation on datasets. These additional cross-validation procedures provided a more comprehensive understanding of the robustness of our model and ensured that our conclusions were not merely fortuitous outcomes based on specific data partitioning.

**Table S2.** 5 fold cross-validation results.

| **Dataset** | **Model** | **Precision** | **Recall** | **F1** |
| --- | --- | --- | --- | --- |
| Matscholar | BioBERT | 88.79 | 90.45 | 90.18 |
|  | SciBERT | 89.46 | 90.64 | 90.04 |
|  | MatSciBERT | 89.30 | 91.23 | 90.25 |
|  | MatSciBERT-Softmax | 89.31 | 91.28 | 90.29 |
|  | MatSciBERT-CRF | 90.26 | 91.64 | 90.95 |
|  | MatSciBERT-BiLSTM-CRF | 90.33 | **91.93** | 91.12 |
|  | MatSciBERT-MRC | **92.21** | 90.80 | **91.49** |
| BC4CHEMD | BioBERT | 92.67 | 95.31 | 93.97 |
|  | SciBERT | 92.52 | 95.33 | 93.90 |
|  | MatSciBERT | 93.52 | 94.77 | 94.19 |
|  | MatSciBERT-Softmax | 93.88 | 94.85 | 94.36 |
|  | MatSciBERT-CRF | 93.47 | 95.49 | 94.47 |
|  | MatSciBERT-BiLSTM-CRF | 93.93 | **95.66** | 94.79 |
|  | MatSciBERT-MRC | **94.51** | 95.33 | **94.92** |
| NLMChem | BioBERT | 92.34 | 93.75 | 92.84 |
|  | SciBERT | 92.54 | 94.96 | 93.73 |
|  | MatSciBERT | 93.21 | 94.98 | 94.09 |
|  | MatSciBERT-Softmax | 93.81 | 94.78 | 94.29 |
|  | MatSciBERT-CRF | 94.41 | 95.12 | 94.92 |
|  | MatSciBERT-BiLSTM-CRF | 94.22 | 94.96 | 94.68 |
|  | MatSciBERT-MRC | **95.96** | **96.39** | **96.18** |
| SOFC | BioBERT | 81.71 | 89.72 | 85.52 |
|  | SciBERT | 82.89 | 88.54 | 85.62 |
|  | MatSciBERT | 82.91 | 89.03 | 85.85 |
|  | MatSciBERT-Softmax | 82.86 | 89.02 | 85.82 |
|  | MatSciBERT-CRF | 85.06 | 88.76 | 86.86 |
|  | MatSciBERT-BiLSTM-CRF | 84.61 | 88.74 | 86.62 |
|  | MatSciBERT-MRC | **86.21** | **89.36** | **87.75** |
| SOFC-Slot | BioBERT | 69.79 | 74.49 | 72.12 |
|  | SciBERT | 70.32 | 74.95 | 72.52 |
|  | MatSciBERT | 70.07 | 76.03 | 72.91 |
|  | MatSciBERT-Softmax | 70.09 | 77.46 | 73.38 |
|  | MatSciBERT-CRF | 72.30 | 77.71 | 74.91 |
|  | MatSciBERT-BiLSTM-CRF | 72.72 | 76.59 | 74.57 |
|  | MatSciBERT-MRC | **82.81** | **81.75** | **81.75** |
